# 

**Published:** 1843-04-01

**Authors:** 


### BIBLIOGRAPHICAL RECORD.

1. The British Journal of Homœopathy. Published quarterly. Heath, St. Paul's Church-yard. 1843.

---

2. Les Bains de Brousse (en Bithyme, Turque d'Asie), avec une Vue des Bains, &c. &c. Par C. A. Bernard, M.D. Constantinople. 1842.

---

3. Cases of Peritoneal Section for the Extirpation of Diseased Ovaria, by the large Incision from Sternum to Pubes, successfully treated. By Charles Clay, M.R.C.S. &c. From the Medical Times. Quarto. Munro and Congreve. 1842.

---

4. Extracts from an unpublished Work on the Importation and Propagation of Plague and other Contagious Diseases. By Dr. Ferguson, Inspector-General of Army Hospitals. (From the Edinburgh Medical and Surgical Journals.)

---

5. On Gravel, Calculus, and Gout, chiefly an Application of Professor Liebig's Physiology to the Prevention and Cure of these Diseases. By H. Bence Jones, M.A. Cantab. &c. Octavo, pp. 142. Taylor and Walton, London. 1842.

---

6. Practical Observations in Midwifery, with Illustratlons. By John Ramsbotham, M.D. &c. Second Edition, revised, in one volume. Highley, 1843.

7. The Physical Diagnosis of Diseases of the Lungs. By Walter Hayle Walshe, M.D., Professor of Pathological Anatomy in University College, London, &c. Small 8vo, pp. 307. Taylor and Walton, 1842.

---

8. Hippopathology; the Diseases of the Horse—Brain, Nerves, and Eyes. Part I. Vol. III. By William Percival, M.R.C.S. Veterinary Surgeon to the 1st Life Guards, &c. Octavo, pp. 158. Longman and Co. 1843.

---

9. The Annals of Chemistry and Practical Pharmacy, Vol. I. No. 15. Published every Friday Morning. Longman and Co. 1843. Price 8d.

---

10. Selections in Pathology and Surgery; or, an Exposition of the Nature and Treatment of Local Diseases, exhibiting new Pathological Views, &c. &c. Illustrated by Cases. By John Davies, M.D. Surgeon to the General Infirmary, Hertford. 8vo. Second Edition. Longman and Co. 1843.

---

11. A System of Clinical Medicine. By Robert James Graves, M.D., one of the Physicians of the Meath Hospital, &c. &c. Octavo, pp. 638. Fannin and Co., Dublin. 1843.

12. Outlines of Pathology and Practical Medicine. By W. Pulteney Alison, M.D. Part I. Preliminary Observations. Part II. Inflammatory and Febrile Diseases. 1 Vol. 8vo, pp. 498. Blackwood, Edinburgh, and Pall-mall, London. January, 1843.

---

13. On Diseases of the Bladder and Prostate Gland. With Plates. Third Edition, revised and corrected. By William Coulson. Longman and Co. Jan. 1843.

---

14. Appendix to the Reports regarding the Royal Infirmary of Edinburgh, 1841—and also for 1842. Compiled by Thomas Bevel Peacock, M.D.

☞ *These Reports, entirely tabular, appear to be carefully and ably prepared by Dr. Peacock.*

---

15. Observations on the principal Medical Institutions and Practice of France, Italy, and Germany, with Notices, &c. By Edwin Lee, M.R.C.S. Second Edition, 1843. Churchill, London.

---

16. The New England Quarterly Journal of Medicine and Surgery. Edited by Drs. Chas. E. Ware, and Samuel Parkman. Boston, July and October, 1842. Pp. 158 each number.

☞ *We hail with pleasure this new contemporary, which appears to be ably conducted. We shall notice some of the articles in our Periscope.*

---

17. Views upon the Statics of the Human Chest, Animal Heat, and Determination of Blood to the Head. By Julius Jeffreys, F.R.S. &c. Octavo, pp. 223. Longman and Co. 1843.

---

18. Two Lectures on the defective Arrangements in large Towns to secure the Health and Comfort of their Inhabitants, &c. By Humphry Sandwith, M.D. 8vo, sewed, One Shilling. Simpkin and Marshall. 1843.

---

19. Observations on the Extraction of Teeth. By Chitty Glendon, Surgeon-Dentist. Duodecimo. Highley, 1843.

---

20. Pharmacologia; being an extended Inquiry into the Operations of Medicinal Bodies, upon which are founded the Theory and Art of Prescribing. By J. A. Paris, M.D. &c. Ninth Edition, re-written. 8vo, pp. 622. Highley, 1843.

---

21. Criminal Jurisprudence considered in relation to Cerebral Organization. By M. B. Sampson. Second Edition. Highley, 1843. Pp. 147.

---

22. A Guide to the Urinary Cabinet: being concise Directions for a Chemico-Pathological Examination of the Urine and Urinary Concretions, &c. &c. By Robert Venables, M.D. &c.

☞ *This Urinary Cabinet, containing the instruments, necessary tests, &c., may be seen at Messrs. Knight and Sons, Foster Lane, London.*

---

23. Interment and Disinterment; or a further Exposition of the Practices pursued in the Metropolitan Places of Sepulture; and the Results as affecting the Health of the Living, &c. By G. A. Walker, Surg. (Re-printed from the Morning Herald.) Octavo, pp. 28. London: Longman and Co. 1843.

---

24. Martin's Thames and Metropolis Improvement Plan; the Objects being to supply the Metropolis with Pure Water—to embank the River, &c. Octavo, pp. 52. Ridgway, London. Feb. 1843.

---

25. The Cold-Water System, an Essay, &c. &c. By Thomas J. Graham, M.D. Octavo, pp. 168. Simpkin and Marshall. Feb. 1843.

---

26. On the Preparations of the Indian Hemp, or Gunjah (Cannabis Indica), their Effects on the Animal System in Health and Disease, &c. By W. B. O'Shaughnessy, M.D., Bengal Army. 1843.

---

27. Metropolis; Summary of the Weekly Tables of the Mortality for 1842.

---

28. Mesmerism; its Pretensions and Effects upon Society considered. By John Brown, M.D. &c. Boston, (Lancashire.) Feb. 1843. Eight Pages.

---

29. A Treatise on Diet; comprising the Natural History, Properties, Composition, Adulteration, and Uses of Vegetables, Animals, Fishes, &c., used as Food. By Wm. Davidson, M.D. &c., lately Senior Physician to the Glasgow Infirmary, &c. 8vo, pp. 383. Churchill, London. March, 1843.

---

30. Popular Cyclopœdia of Natural Science, Mechanical Philosophy, and its Application to the Arts. By William B. Carpenter, M.D. &c. Part III. Price 4s. W. S. Orr and Co. 1843.

---

31. Observations on an Act (5 and 6 Victoria, c. 123) for amending the Law relating to Private Lunatic Asylums in Ireland, &c. With Comments, &c. By Wm. Harty, M.D., Physician to the Prisons of Dublin. Pp. 49. Dublin. March, 1843.

---

32. Dublin Journal of Medical Science. No. 67. March, 1843.

---

33. The Physiological Anatomy and Physiology of Man. By Robert Bentley Todd, M.D. F.R.S. &c., and William Bowman, F.R.S., Assistant Surgeon to King's College Hospital, &c. Part the First, 1843. Parker, West Strand. Price 7s. each Part.

---

34. Annals of Chemistry and Practical Pharmacy, for March, 1843. Price One Shilling.

---

35. A concise Historical Sketch of the Progress of Pharmacy in Great Britain. Intended as an Introduction to the Pharmaceutical Journal. By Jacob Bell. Price One Shilling. Churchill, London. March, 1843.

---

36. The Naturalist's Library, conducted by Sir W. Jardine, Bart. Ichthyology. Vol. IV. British Fishes, Vol. I. By Robert Hamilton, M.D. &c. Edinb. Lizars. London, Highley. 1842.

---

37. A new Theory and Treatment of Disease, founded upon Natural Principles. By John Tennion, M.D. Machlachlan and Stewart, Edinb. 1843. Pp. 35.

---

38. Animal Magnetism and Homœopathy, with Notes, &c. By Ed. Lee, Esq. Third Edition.

☞ *Mr. Lee has wheeled round and embraced Mesmerism, after writing against it!! We need say no more!*

---

39. On the Preparation of Extracts by Spontaneous Evaporation, assisted by a Current of Dry Air. By Mr. W. Hooper, of Pall-mall East. *(Read before the Pharmaceutical Society.)*

☞ *The apparatus for preparing these extracts cannot be described in words, but is represented by a wood-cut in Mr. Hooper's ingenious communication.*

---

40. Nineteenth Annual Report of the Visitors of the General Lunatic Asylum, County and City of Gloucester. 1842.

---

41. Fourth Annual Report of the Registrar-General of Births, Deaths, and Marriages in England. 8vo, pp. 361. Clowes, 1842.

---

42. The Life of a Travelling Physician, from his first Introduction to Practice; including Twenty Years' Wandering, &c. &c. Three Vols. 8vo. Longman & Co. 1843.

